# Plasticity changes in forebrain activity and functional connectivity during neuropathic pain development in rats with sciatic spared nerve injury

**DOI:** 10.1186/s13041-018-0398-z

**Published:** 2018-10-01

**Authors:** Tzu-Hao Harry Chao, Jyh-Horng Chen, Chen-Tung Yen

**Affiliations:** 1 Department of Life Science, National Taiwan University, No. 1, Sec. 4, Roosevelt Rd, Taipei, 10617 Taiwan; 2 Interdisciplinary MRI/MRS Lab, Department of Electrical Engineering, National Taiwan University, No. 1, Sec. 4, Roosevelt Rd, Taipei, 10617 Taiwan

**Keywords:** Neuropathic pain, Chronic pain, Plasticity, fMRI, MEMRI, Unit recording, Insular cortex

## Abstract

Neuropathic pain is a major worldwide health problem. Although central sensitization has been reported in well-established neuropathic conditions, information on the acute brain activation patterns in response to peripheral nerve injury is lacking. This study first mapped the brain activity in rats immediately following spared nerve injury (SNI) of the sciatic nerve. Using blood-oxygenation-level-dependent functional magnetic resonance imaging (BOLD-fMRI), we observed sustained activation in the bilateral insular cortices (ICs), primary somatosensory cortex (S1), and cingulate cortex. Second, this study sought to link this sustained activation pattern with brain sensitization. Using manganese-enhanced magnetic resonance imaging (MEMRI), we observed enhanced activity in the ipsilateral anterior IC (AIC) in free-moving SNI rats on Days 1 and 8 post-SNI. Furthermore, enhanced functional connectivity between the ipsilateral AIC, bilateral rostral AIC, and S1 was observed on Day 8 post-SNI. Chronic electrophysiological recording experiments were conducted to confirm the tonic neuronal activation in selected brain regions. Our data provide evidence of tonic activation-dependent brain sensitization during neuropathic pain development and offer evidence that the plasticity changes in the IC and S1 may contribute to neuropathic pain development.

## Introduction

Chronic pain is a major health problem affecting up to 20% of the global population. Although acute pain can be properly managed, most people with chronic pain do not have access to adequate pain relief [1]. Among the most difficult cases are those with neuropathic pain initiated by primary lesions or dysfunction in the somatosensory nervous system.

Many studies have investigated brain sensitization in well-established chronic neuropathic conditions. Higher tonic activity or sensitization to peripheral and central stimuli have been detected in many brain regions of humans with such conditions [2–4] and in animal models [5, 6]. Central sensitization at the spinal level was reported as activation-dependent and possibly induced by repeated stimulation [7–10]. In addition, some brain areas have been reported as having similar activation-dependent sensitization properties [11, 12]. By investigating the responses to nerve injury in the peripheral and central brain areas, previous studies have detected the early onset of ectopic discharge in injured nerve fibers [13–15]. One study of trigeminal neuropathic pain in rats showed that nerve injury induced the long-term enhancement of activity in trigeminal ganglion neurons and thalamic neurons [15]. Although strong and continuous brain activation is a possible cause of brain sensitization during neuropathic pain development, experimental data are lacking.

Practically, the study of brain reactions to severe peripheral nerve injury in human subjects is difficult. Therefore, human subjects for such studies are usually selected from people who have received a diagnosis of chronic pain [2, 16, 17]. By contrast, using animal models enables researchers to study brain reactions immediately following peripheral nerve injury [18, 19].

To observe the dynamic changes of brain activity during nerve injury and the possible sustained activation, we designed a nerve cutting device for inducing spared nerve injury (SNI) in the functional magnetic resonance imaging (fMRI) chamber. We used a recoverable anesthesia protocol during blood-oxygenation-level-dependent (BOLD)-fMRI [20–23], and thus could observe neuropathic pain development in the same rat after the BOLD-fMRI experiment.

To correlate the sustained activation with long-term plasticity changes in the brain, we used manganese-enhanced magnetic resonance imaging (MEMRI) to detect plasticity changes in brain activity on Days 1 and 8 after SNI. Recent studies have used MEMRI to assess brain activity in free-moving animals [24–26]. Mn^2+^ can accumulate in excitable cells via voltage-gated Ca^2+^ channels and ionotropic glutamate receptors [27–29], thereby enhancing the signal in T1 contrast images and providing high spatial resolution mapping of brain activations.

Finally, we confirmed the tonic neuronal activations observed in the MR with direct neuronal recording in the insula cortices (ICs) and primary somatosensory cortex (S1). Sustained brain activations induced by SNI were observed by resting fMRI and electrophysiological recording, and these changes correlated with long-term brain plasticity in rats with neuropathic pain.

## Methods

### Animal subjects

This study used 55 male and 15 female Sprague Dawley rats (National Laboratory Animal Center, Taipei, Taiwan) aged 8–10 weeks and weighing 250–350 g. Vendor health reports indicated that the rats were free of known viruses, bacteria, and parasites. All the rats were housed pairwise in type 3H cages under a 12-h dark/light cycle with an environmental temperature of 22 °C. The cages were filled with C-grade Sani-Chips and food and water were available ad libitum. All experimental procedures were approved by the Institutional Animal Care and Use Committee of National Taiwan University. This study adhered to the guidelines established by the Council of Agriculture of Taiwan for the experimental use of animals.

**Fig. 1:**
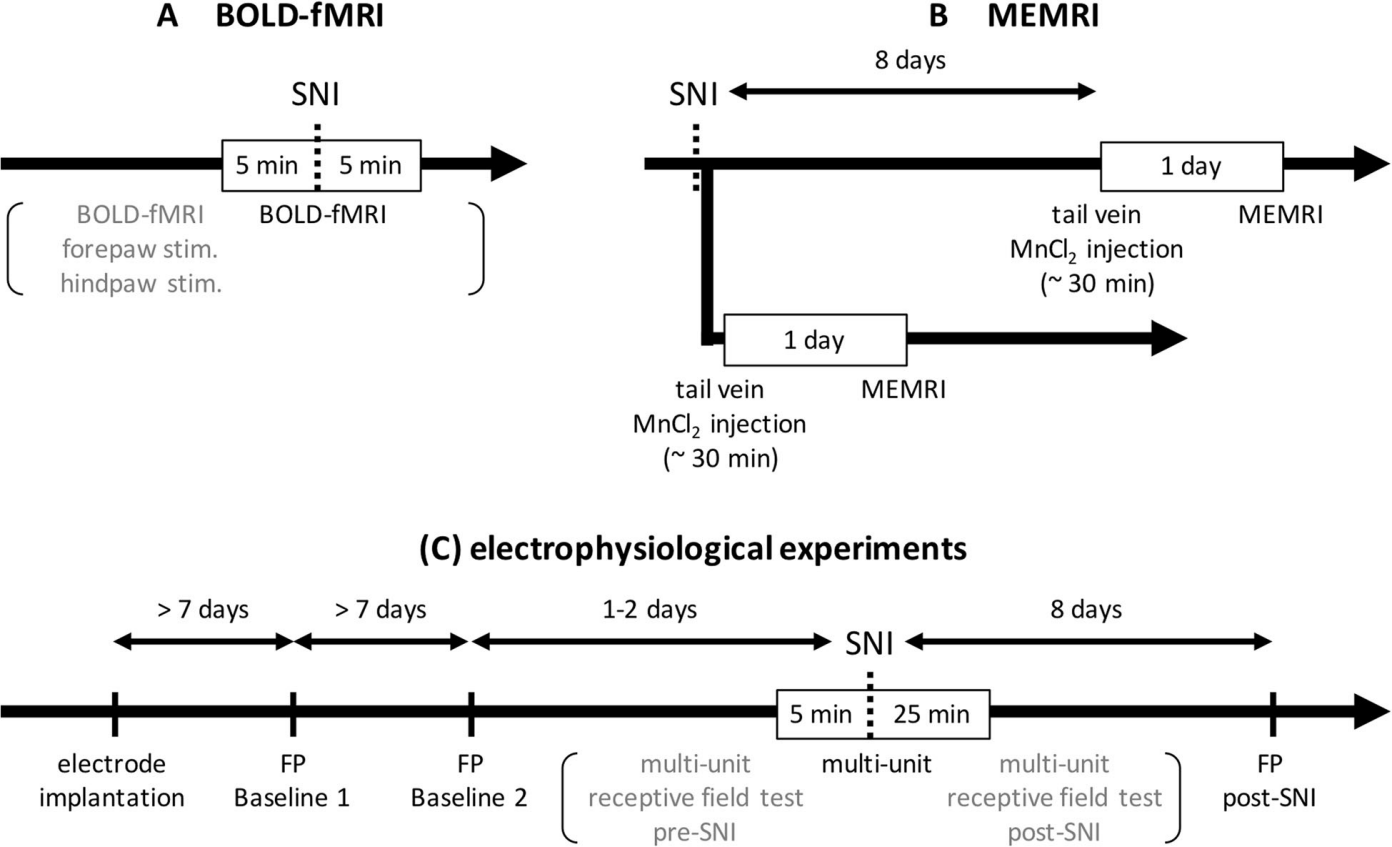
Experimental design in this study. (**a**) The experimental design of BOLD-fMRI study. Rats were anesthetized by continuous intravenous infusion of dexmedetomidine (0.05 mg/kg/h) and rocuronium bromide (9 mg/kg/h). Before the formal fMRI study of the brain response to SNI, forepaw and hind paw stimulation-evoked BOLD responses were confirmed twice to ensure that the rats’ physiological conditions were suitable for fMRI and to confirm that the sciatic nerve was functionally intact. After confirmation, a 10-min fMRI session was conducted (yielding 300 images) with nerve transection at the 151st scan. (**b**) The experimental design of MEMRI study. The rats were divided into two groups. The purpose of the first group was to understand the cumulative brain activity during the first 24 h after neuropathic pain initiation, whereas that of the second group was to understand the cumulative brain activity on Day 8 after neuropathic pain initiation. The rats in the first group were anesthetized by isoflurane for SNI surgery. After SNI, a saline solution of 120 mM MnCl_2_ (75 mg/kg; 2.25 mL/h) was injected into the tail vein. MEMRI scanning was performed 24 h after MnCl_2_ infusion under 2% isoflurane anesthesia. The rats in the second group initially received SNI surgery under anesthesia by ketamine hydrochloride (75 mg/kg, i.p.) and xylazine (15 mg/kg i.p.). The rats received MnCl_2_ infusion under isoflurane anesthesia 1 week later, and MEMRI scanning was performed 24 h after MnCl_2_ infusion under 2% isoflurane anesthesia. (**c**) The experimental design of electrophysiology study. One week after electrode implantation, the EFP of the thalamocortical pathway was recorded twice before SNI for a baseline system stability check under 1.5–2% isoflurane anesthesia. Subsequently, each rat received SNI under the same anesthesia protocol as that of the BOLD-fMRI experiment, dexmedetomidine (0.05 mg/kg/h) and rocuronium bromide (9 mg/kg/h). During SNI, multiunit activities were recorded from 5 min before SNI to 25 min after SNI

Seven rats (four males and three females) were used in the BOLD-fMRI study of dynamic brain activity changes during nerve injury. In the MEMRI study of long-lasting changes of brain activity in free-moving rats, the sham (*n* = 10) and SNI (*n* = 10) groups were observed on Day 1 and additional independent sham (n = 10) and SNI (*n* = 8) groups were observed on Day 8 after SNI surgery. At both time points, the naïve (*n* = 10) group was observed. Three additional rats were used to validate the adverse behavioral effects of Mn^2+^ injection. All the rats in the MEMRI study were male. The electrophysiological study observed the following four groups of rats: ipsilateral rostral anterior insular cortex (RAIC), contralateral RAIC, contralateral anterior cingulate cortex (ACC), and contralateral S1. Each group consisted of three rats. Because skull growth in male rats is relatively rapid, which likely affects electrophysiological recording quality, we used female rats in our electrophysiological study. The BOLD-fMRI data (mixed-gender) revealed similar result when comparing with the electrophysiological data (female only) in acute response to the nerve injury. The basic design of this study is diagrammatically shown in the Fig. 1.

### SNI neuropathic pain model

The SNI neuropathic pain model used followed the one established by Decosterd and Woolf [30]. Briefly, rats were anesthetized using ketamine hydrochloride (75 mg/kg, i.p.) and xylazine (15 mg/kg i.p.). An incision was made on the left lateral thigh to expose the sciatic nerve at the level of its trifurcation into the sural, tibial, and common peroneal nerves. The left tibial and common peroneal nerves were tightly ligated using 6.0 silk. Two to 4 mm of the distal nerve was removed and the sural nerve was left intact. After nerve transection, we sutured the muscle by using 6.0 silk and closed the incision with 4.0 silk. Subsequently, the rats were recovered from anesthesia and their allodynia behavior was observed 8 days later. The rats received lincomycin (Lita Pharmacy Co. Ltd., Taiwan; 30 mg/kg/day, i.m. for 3 days) after the surgery to prevent infection.

### Behavioral testing

To determine whether the rats had developed neuropathic pain after SNI surgery, we assessed the mechanical allodynia through von Frey filament (North Coast Medical, Inc., Morgan Hill, USA) testing. All behavioral tests were conducted between12:00 and 18:00. Each rat was tested in a 21 × 12 × 14-cm transparent cage after 5 min of habituation. For the mechanical sensitivity test, each rat was placed in a cage with an open wire mesh base and given 15 min for habituation. A set of eight von Frey filaments (0.4, 0.6, 1, 2, 4, 6, 8, and 15 g of bending force) were used to apply increasing amounts of bending force to the lateral plantar surface (the lateral plantar surface was innervated by the sural nerve). If the rat briskly withdrew or one or both of its hind paws flinched, the final lightest von Frey hair force was applied in the subsequent test. Six stimulations were applied in each session and the withdrawal patterns were recorded to determine the 50% withdrawal threshold according to the formula established by Chaplan [31].

### Rat preparation for BOLD-fMRI study

**Fig. 2:**
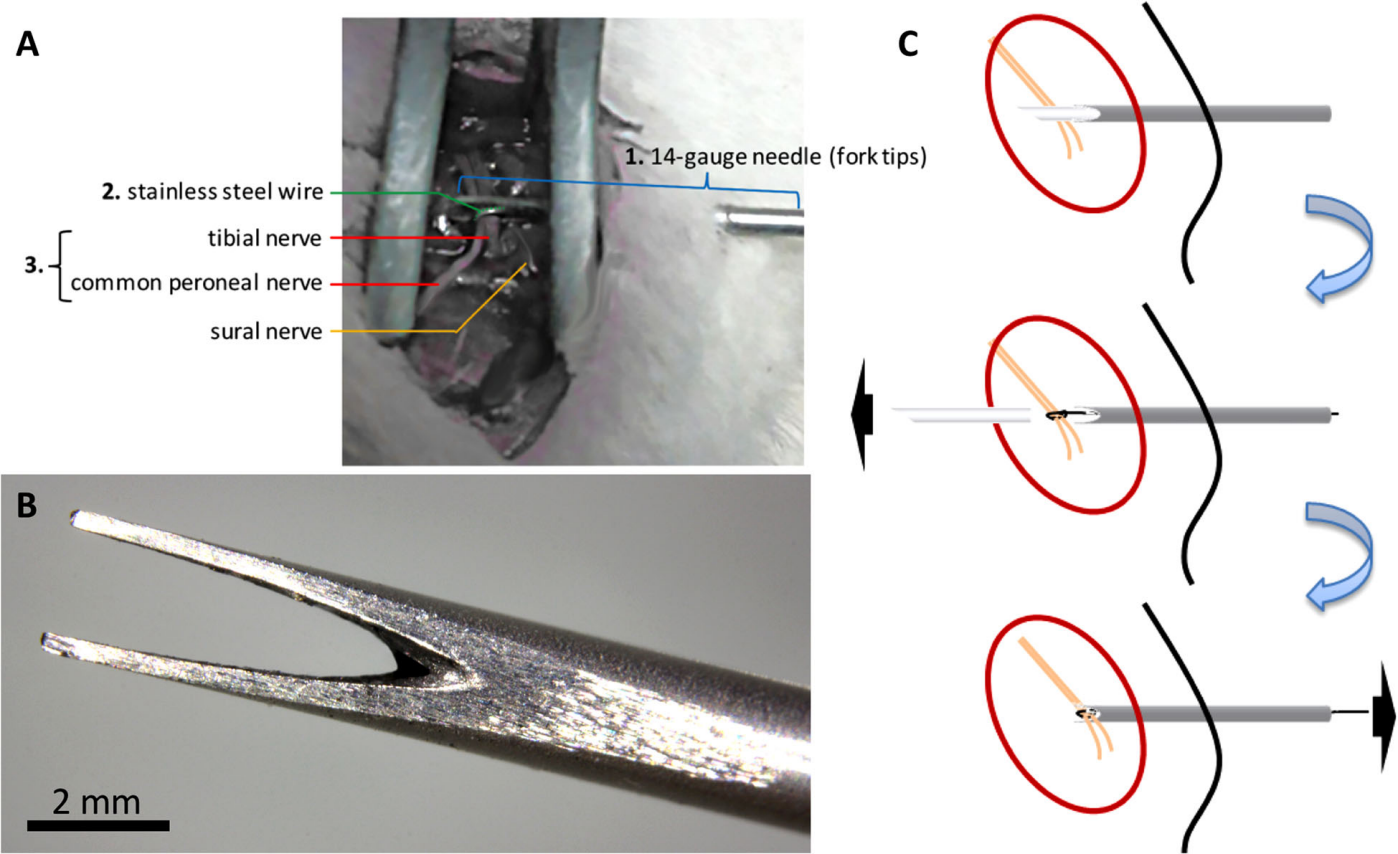
Nerve transection device. (**a**) Photograph of the nerve transection device, which consists of a guidance head and needle with fork tips, and was implanted before MRI scanning (A-1). A stainless steel wire (A-2) was threaded through the needle, and fixed the common peroneal and tibial nerves (A-3) to the notches of the fork tips by using a slack noose. (**b**) Tip of the nerve transection device. Notably, the notch of the tip was polished to the sharpness of a knife to cut the sciatic nerve. (**c**) The device consisted of a 19-gauge guidance needle connected to a 14-gauge needle with fork tips and was inserted at the caudal surface of the ipsilateral thigh through the muscle layers to the sciatic nerve branches. After removal of the guidance head, the fork tips were exposed. A stainless steel wire was threaded through the needle and fixed to the common peroneal and tibial nerves on the notch of the fork tips with a slack noose

All fMRI experiments were conducted between 12:00 and 18:00. To identify the tonic brain activation after nerve injury, SNI surgery was performed inside the MRI bore during fMRI acquisition. A specially designed nerve transection device consisting of a guidance head and needle with fork tips was implanted before MRI scanning (Fig. 2). Each rat was initially anesthetized using 5% isoflurane and orotracheally intubated for mechanical ventilation. Under isoflurane anesthesia (3.5% mixed with a 30% oxygen and 70% nitrogen mixture), a 1-cm-long incision was made on the lateral surface of the left thigh and the biceps femoris muscle was dissected to expose the sural, common peroneal, and tibial sciatic nerve branches. The nerve transection device was inserted at the caudal surface of the left thigh, through the muscle layers to the sciatic nerve branches. After the guidance head had been removed, the fork tips were exposed. A stainless steel wire was threaded through the needle and the common peroneal and tibial nerves were fixed to the notches of the fork tips by using a slack noose (Fig. 2). Finally, lidocaine hydrochloride jelly USP 2% (Akorn, Inc., Lake Forest, IL, USA) was applied on the wound carefully without contacting sciatic nerve branches, and then the incision was temporally sutured. This setup enabled the cutting of the common peroneal and tibial nerves during fMRI scanning by pulling on the stainless steel wire. In addition, a set of bipolar stainless steel electrodes each was inserted between the second and third digits of both the left forepaw and left hind paw, respectively. We used forepaw stimulation to induce S1 BOLD responses to confirm the suitability of the conditions for fMRI testing and hind paw stimulation to induce S1 BOLD responses in order to confirm that our settings did not damage the sciatic nerve before nerve transection.

### BOLD-fMRI acquisition

Imaging data were acquired using a 7-Tesla scanner with a 30-cm diameter bore (Bruker Biospec 7030 USR, Ettlingen, Germany). The system was equipped with a 670-mT/m (175-μs rise time) actively shielded gradient system (Bruker, BGA12-S) with an inner diameter of 116 mm. A receive-only four-element phased array coil was used to receive radio frequency signals and a linear volume coil was used to transmit radio frequency pulses.

After the rats were secured on the MRI holder, a bolus of dexmedetomidine (0.5 mL; 0.025 mg/kg; Dexdormitor, Orion, Espoo, Finland) was injected into the tail vein. Fifteen minutes after the bolus injection, continuous intravenous infusion of dexmedetomidine (1 mL/h; 0.05 mg/kg/h) and rocuronium bromide (9 mg/kg/h; Sigma–Aldrich, St. Louis, USA) was initiated and the isoflurane concentration was adjusted to 0.5–1% for the entire scanning period [20]. During the MRI scan, rectal temperature was measured using a thermocouple (Model 1025, SA Instruments, Inc., New York, USA) and maintained at 36.5–37.5 °C by using a circulated hot water bed. The end-tidal CO_2_ level was continuously monitored and adjusted to between 2.5–3.0%, a range previously calibrated for invasive blood gas sampling under identical conditions to ensure normal physiological conditions in the rat.

The anatomical images were acquired using a rapid acquisition with relaxation enhancement (RARE) sequence (10 coronal slices, thickness = 1 mm, repetition time (TR) = 2500 ms, echo time (TE) = 33 ms, matrix size = 160 × 160, field-of-view (FOV) = 25 × 25 mm, average = 2). To improve the magnetic field homogeneity of the acquisition site, local shimming of the brain area was performed before BOLD-fMRI data acquisition (Mapshim; Bruker BioSpin). BOLD-fMRI data were acquired using single-shot gradient-echo echo planar imaging (EPI) (10 coronal slices, thickness = 1 mm, TR = 2000 ms, TE = 22 ms, matrix size = 80 × 80, FOV = 25 × 25 mm, bandwidth = 200 kHz).

For standard forepaw and hind paw stimulation fMRI, an isolated stimulator (S48 Square Pulse Stimulator with Stimulus Isolation Unit, Grass Technologies, West Warwick, USA) was used to deliver constant current pulses to the stainless steel electrodes for forepaw and hind paw stimulation. These constant current pulses consisted of monophasic square wave electrical stimulation (0.5 ms, 2 mA, 9 Hz) divided into five blocks of 20-s on/off cycles. Ten dummy scans and ten additional baseline images were acquired, yielding 120 images in total. After the nerve was confirmed to be healthy, a 10-min fMRI session was conducted (yielding 300 images) with nerve transection at the 151st scan. After fMRI, we sutured the muscle using 6.0 silk and closed the incision with 4.0 silk. Subsequently, the rat was recovered from anesthesia and paralysis by receiving atipamezole hydrochloride (3 mg/kg, i.v.; ANTISEDAN, Orion, Espoo, Finland), for the reversal of the sedative and analgesic effects of dexmedetomidine, and sugammadex sodium (4–8 mg/kg, i.v.; Merck Sharp & Dohme Corp., Kenilworth, NJ, USA), for the reversal of the paralytic effect of rocuronium, and its allodynia behavior was tested 8 days later. The rat was administered lincomycin (Lita Pharmacy Co. Ltd., Taichung, Taiwan; 30 mg/kg/day, i.m. for 3 days) after scanning to prevent infection.

### Electrode implantation and recordings

Each rat was anesthetized with sodium pentobarbital (50 mg/kg, i.p.), of which supplemental doses (16 mg/kg, i.p.) were administered when necessary. Craniotomies were performed to expose the brain surface vertical to the recording sites. The coordinates for implantation were as follows: (1) mediodorsal thalamic nucleus, medial part (MDM): right or left: 0.4 mm, posterior: 3 mm, depth: 4.5–5.5 mm; (2) rostral anterior insular cortex (RAIC): right or left: 3–5 mm, anterior: 1–4 mm, depth: 5–6 mm; (3) mediodorsal thalamic nucleus, lateral part (MDL): right: 1.2 mm, posterior: 2.5 mm, depth: 4.5–5.5 mm; (4) anterior cingulate cortex (ACC): right: 1.2 mm, anterior: 1–4 mm, depth: 2.5–3 mm; (5) ventral posterolateral thalamic nucleus (VP): right: 2.8–3.3 mm, posterior: 2.8 mm, depth: 5.5–6.5 mm; (6) S1: right: 1–3 mm, posterior: 1 mm, depth: 600–800 μm. For thalamus recording, we used a bundled microarray electrode consisting of seven tungsten microwires with diameters of 35 μm bare and 50 μm insulated (#100211; California Fine Wire) in a 29-G guide tube [32]. The electrode set for the cortex recording consisted of eight stainless steel microwires arranged in a 2–3-mm-wide array. Each rat was implanted with a matching thalamic and cortical target set (MDM-RAIC, MDL-ACC and VP-SI) of electrodes. Only those rats with good evoked cortical responses under low intensity (below 10 μA) thalamic stimulation were used to ensure the accuracy of the cortical implantations. Four stainless steel screws were set in each rat’s skull to serve as anchors for the electrode sets. To ground the array electrodes, a copper wire was fixed around the anchoring screw positioned at the occipital bone. When all the electrodes were in place, the surface of the skull was covered with dental cement and the wound was sutured. Lincomycin hydrochloride (30 mg/kg, i.m.) was administrated to prevent infection.

After recovering for 1 week, single unit activities were recorded while SNI surgery was conducted. Multiple-channel cortical unit activities were transmitted to a multichannel acquisition processor system (MAP, Plexon, Dallas, USA) through a connecting cable. To record single unit activities while performing SNI surgery, the anesthesia procedure and surgery preparation were identical to those of the fMRI experiment. Single unit recording was initiated 5 min before sciatic nerve transection and continued for 25 min after transection, resulting in 30 min of continuous recording. Spike signals were amplified 7000–32,000-fold, bandpass-filtered at 250 Hz–13 kHz, and digitized at 40 kHz. Well isolated single unit activities in one area were linearly added for a quantitative estimation of the activity change in that cortical area.

### Animal preparation for MEMRI study

Two groups of rats underwent MEMRI testing. The first group was tested to determine the cumulative brain activity during the first 24 h of neuropathic pain initiation, whereas the second group was tested to determine the brain activity on Day 8 following neuropathic pain initiation.

Each rat in the first group was initially anesthetized using 5% isoflurane and maintained in a state of anesthesia by using 3.5% isoflurane. SNI surgery was performed as previously described. Following SNI or sham surgery, a solution of 120 mM MnCl_2_ in saline (75 mg/kg; 2.25 mL/h; MnCl_2_-4H_2_O, Sigma–Aldrich, St. Louis, USA) was injected into the tail vein. The MnCl_2_ injection protocol followed that of a previous study [33]. During surgery, each rat’s body temperature was maintained at 36.5–37.5 °C by using a feedback-controlled heating pad. After surgery, each rat was administered lincomycin (30 mg/kg/day, i.m. for 3 days) to prevent infection. MEMRI scanning was performed 24 h after MnCl_2_ infusion and each rat’s allodynia behavior was observed on Days 3 and 8 after scanning.

Following our previous study, each rat in the second group underwent initial SNI or sham surgery under ketamine hydrochloride anesthesia (75 mg/kg, i.p.) and xylazine (15 mg/kg i.p.) [34]. Lincomycin (30 mg/kg/day, i.m. for 3 days) was administered after surgery and all rats were tested for allodynia behavior 1 week later. As previously described, rats in which neuropathic pain was observed were administered MnCl_2_ infusion under isoflurane anesthesia and MEMRI scanning was performed 24 h later.

### MEMRI acquisition

Imaging data were acquired using the 7-Tesla scanner. The receive-only four-element-phased array coil and linear volume coil were used to receive radio frequency signals and transmit radio frequency pulses, respectively. Each rat was initially anesthetized by 5% isoflurane mixed with oxygen and maintained in a state of anesthesia under 2% isoflurane during scanning. The T1-weighted images were acquired using a RARE sequence (30 coronal slices, thickness = 0.7 mm, TR = 870 ms, TE = 8.4 ms, matrix size = 160 × 160, FOV = 25 × 25 mm, average = 16). During scanning, each rat’s respiration rate was monitored using a pressure sensor placed below the abdomen. A reading within the range of 40–60 breath/min signified stability. Rectal temperature was measured using a thermocouple and maintained at 36.5–37.5 °C by using a circulated hot water bed.

### Image processing and analysis

Statistical parametric maps were generated using the SPM version 8 (www.fil.ion.ucl.ac.uk/spm). All raw images were enlarged by a factor of ten to correlate the image dimensions to human data, thereby facilitating the use of SPM, which was developed for use on humans. For the fMRI data, EPI images obtained from a single session were realigned and resliced to their averaged image to minimize movement artifacts. The realigned images were further coregistered to the average image between subjects. Subsequently, these images were smoothed using a Gaussian kernel with a full width at half maximum (FWHM) of 8 mm (in the space of the enlarged images) to reduce white noise and blur the images. Individual statistical maps were plotted using a general linear model with a hemodynamic response function. A 0.0078-Hz high-pass filter was applied to remove slow signal drift. Group analysis of individual statistical maps was conducted using a single sample *t* test. The significant BOLD response was determined by an individual voxel threshold of *P* < .02 with a cluster size threshold of 23 continuous voxels (volume = 2.25 mm^3^). According to the AlphaSim procedure [35], this threshold combination provides a probability of false-positive clusters within the brain area of *P* < .05.

In the MEMRI data, the images of each subject were coregistered together and the average image from the coregistered images served as the template image. Subsequently, all MEMRI images were coregistered to the template image again and these fine-aligned images were smoothed using a Gaussian kernel with a FWHM of 3 mm (in the space of the enlarged images). The extra brain area was then removed. Statistical parametric maps were generated and one-way analysis of variance (ANOVA) was performed to compare the groups for differences. Grand mean scaling and global normalization scaling were used and an absolute threshold masking of 0 was applied to exclude the extra brain area from the analysis. Significance was determined by an individual voxel threshold of *P* < .05 with a cluster size threshold of 60 continuous voxels (volume = 1.025 mm^3^). According to the AlphaSim procedure [35], this threshold combination provides a probability of false positive clusters within the brain area of *P* < .03. The colors of significant areas in statistical maps were encoded from statistical *t* values. Warm and cold colors indicate increases and decreases in BOLD (or MEMRI) signals, respectively.

Resting-State fMRI Data Analysis Toolkit V1.6 (http://restfmri.net/forum/index.php) was used to conduct functional connectivity analysis of the fMRI data. For fMRI data acquired before and after SNI, EPI images acquired within the first 4 min (first 120 images) and final 4 min (final 120 images) of the 10-min fMRI session were used to generate correlation maps of the pre- and post-SNI groups, respectively. All preprocessed image series were detrended and bandpass-filtered (0.01–0.08 Hz) before the functional connectivity analysis. The correlation maps were transformed into z-statistical parametric maps using REST, and the pre- and post-SNI groups were compared by conducting a paired *t* test with SPM. The multiple regression analysis function in SPM was used for functional connectivity analysis of the MEMRI images. To determine the brain areas with functional connections to specific regions of interest (ROIs), the inter-subject variability of neural activities in selected ROIs were used as the regressor. These activities were quantified and normalized based on the following equation: average signal intensity within an ROI/average signal intensity of the entire brain.

### Statistical analysis

All data are expressed as the mean ± standard error. A *P* value of <.05 was considered statistically significant. Brain activity in the sham and SNI groups was compared by conducting an independent two-sample *t* test. The results of the hind paw withdrawal threshold for both groups were compared through one-way repeated measures ANOVA and Tukey’s post hoc multiple comparison.

## Results

### Immediate functional brain changes after spared nerve injury surgery

**Fig. 3:**
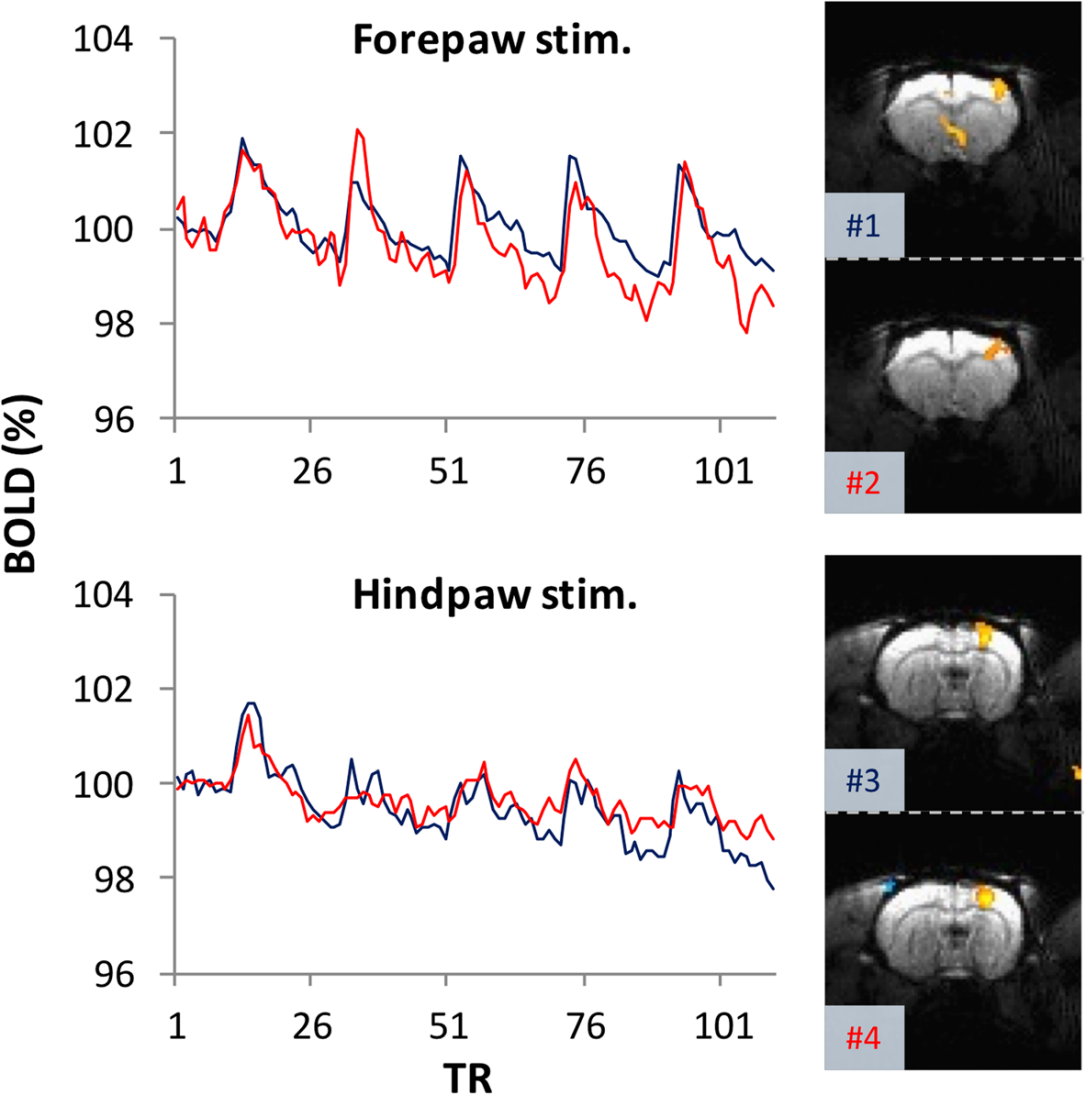
Represented results of fMRI checkpoints before the formal study. Before the fMRI investigation of the brain responses to SNI nerve transection, we used the S1 BOLD response to forepaw stimulation to confirm whether the experimental conditions were suitable for fMRI scanning (upper panel). Subsequently, we used the S1 BOLD response to hind paw stimulation to verify whether the sciatic nerve was intact after implantation of the nerve transection device (lower panel). In the represented data, the S1 forepaw and hind paw areas exhibited consistent spatial and temporal responses to forepaw and hind paw stimulation, respectively, among the successive scans. #1–4 indicate the scan order. TR stands for the repetition time for each acquisition, which was 2 s in the current study

To analyze the acute brain responses to nerve transection and determine the sustained activated brain areas possibly related to neuropathic pain development, we developed a nerve transection device for conducting SNI inside the MRI machine (Fig. 2). Before the formal fMRI experiment of the brain responses to SNI, forepaw and hind paw stimulation–evoked BOLD responses were obtained to confirm the rat’s physiological condition and the healthiness of the sciatic nerve, with the nerve transection devise implanted, respectively. The robust and identical S1 BOLD responses to forepaw stimulation indicated that the rats’ physiological conditions were suitable for the fMRI experiment. The S1 BOLD responses to hind paw stimulation indicated that each rat’s sciatic nerve was functionally intact before SNI (Fig. 3).

**Fig. 4:**
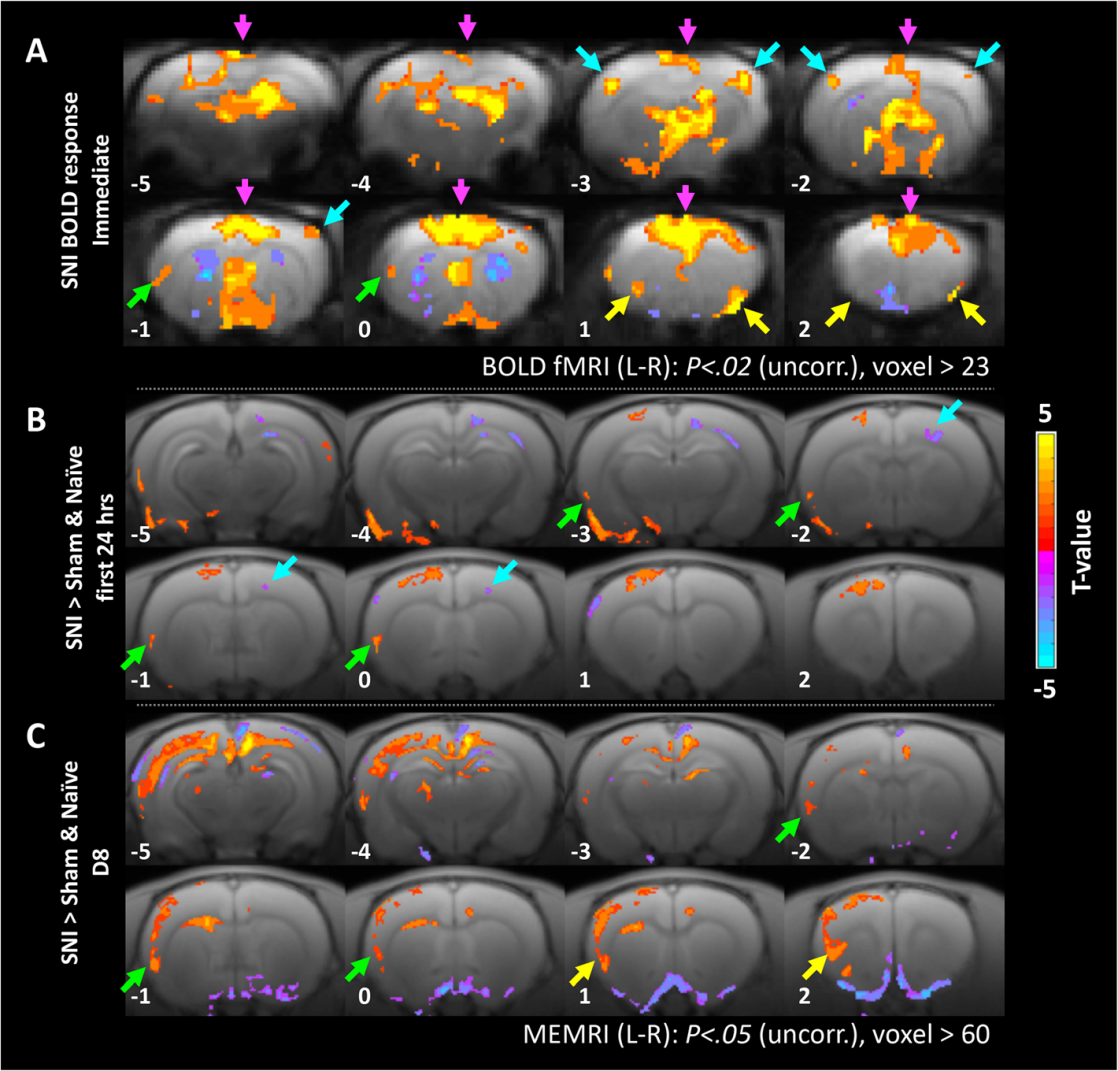
Functional brain images at various time points after SNI. In each sub-figure, statistical t-maps are shown in 8 consecutive coronal slices of the rat brain, 1 mm each, from the most caudal level (5 mm caudal to the bregma, upper-left corner) to the most rostral (2 mm rostral to the bregma, lower-right). (**a**) Brain responses to SNI surgery during fMRI scanning. Positive BOLD responses are shown in the S1HL area (blue arrows), CC (purple arrows), and RAIC (yellow arrows) (*n* = 7, four males and three females). (**b**) MEMRI voxel-wise comparison of brain activity between the SNI (*n* = 10), sham (*n* = 10), and naïve (*n* = 10) rats on Day 1 after SNI surgery. The SNI group exhibited reduced activity in the S1 contralateral to the injured hind limb (blue arrows) and increased activity in the ipsilateral AIC (green arrows). (**c**) MEMRI voxel-wise comparison of brain activity between the SNI (*n* = 10), sham (*n* = 8), and naïve (*n* = 10) rats on Day 8 after SNI surgery. The SNI group exhibited increased activity in the RAIC and AIC (yellow and green arrows, respectively). The SNI and sham groups respectively shown in (**b**) and (**c**) were independent. Note Robust RAIC activation induced immediately after SNI (yellow arrow in **a**). The enhanced RAIC activity was maintained over 8 days (green arrows in **c**)

**Fig. 5:**
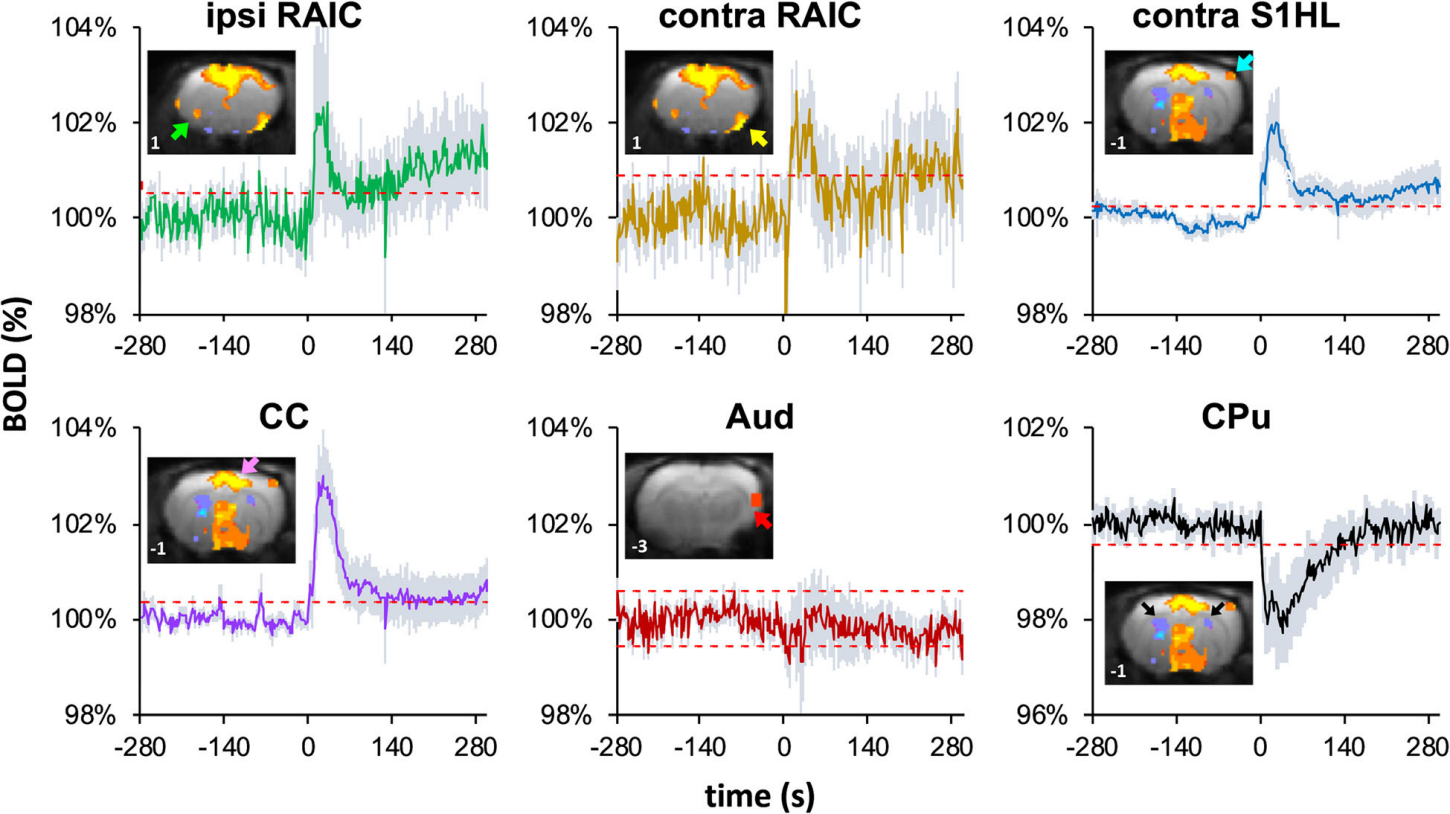
Average time courses of BOLD signals in various brain areas. The average BOLD signals are plotted in dark colors and the standard errors are shown in light blue. All ROIs are labeled with arrows in the insets. The dashed red lines indicate the boundary of |z-score| = 2.33 (*P* < .01). The SNI nerve transection induced a transient positive BOLD response followed by gradually enhanced BOLD signals in the bilateral ICs and S1HL area contralateral to the SNI hind limb, whereas the CC exhibited a robust positive response followed by sustained low level activity. In addition, the CPu exhibited a strong negative BOLD response after nerve transection. The contralateral auditory cortex (Aud) was selected as the control ROI to verify the BOLD signal stability. No evident BOLD signal changes were observed in the Aud after SNI. In these time courses, the first 140 scans (from − 280 to 0 s before nerve transection) show the BOLD signals under the resting baseline condition. SNI nerve transection was conducted upon initiation of the 141st scan (time = 0; *n* = 7)

Transection of the left tibial and common peroneal nerves produced significant positive BOLD responses in the medial thalamus, hypothalamus, and S1 hind limb area (S1HL) contralateral to the injured nerve, and cingulate cortex (CC) and RAICs bilaterally. Significant negative BOLD responses were also found in the bilateral caudate putamen (CPu) (Fig. 4A). Moreover, examining each ROI, the increases in the BOLD signal in the bilateral ICs and contralateral S1HL area were continuously enhanced throughout the acquisition period after nerve transection (more than 5 min); the CC exhibited a sustained low level of activation; whereas the CPu showed a robust transient negative BOLD response. The auditory cortex (Aud), selected as a negative control, didn’t show significant response during the nerve transection (Fig. 5).

**Fig. 6:**
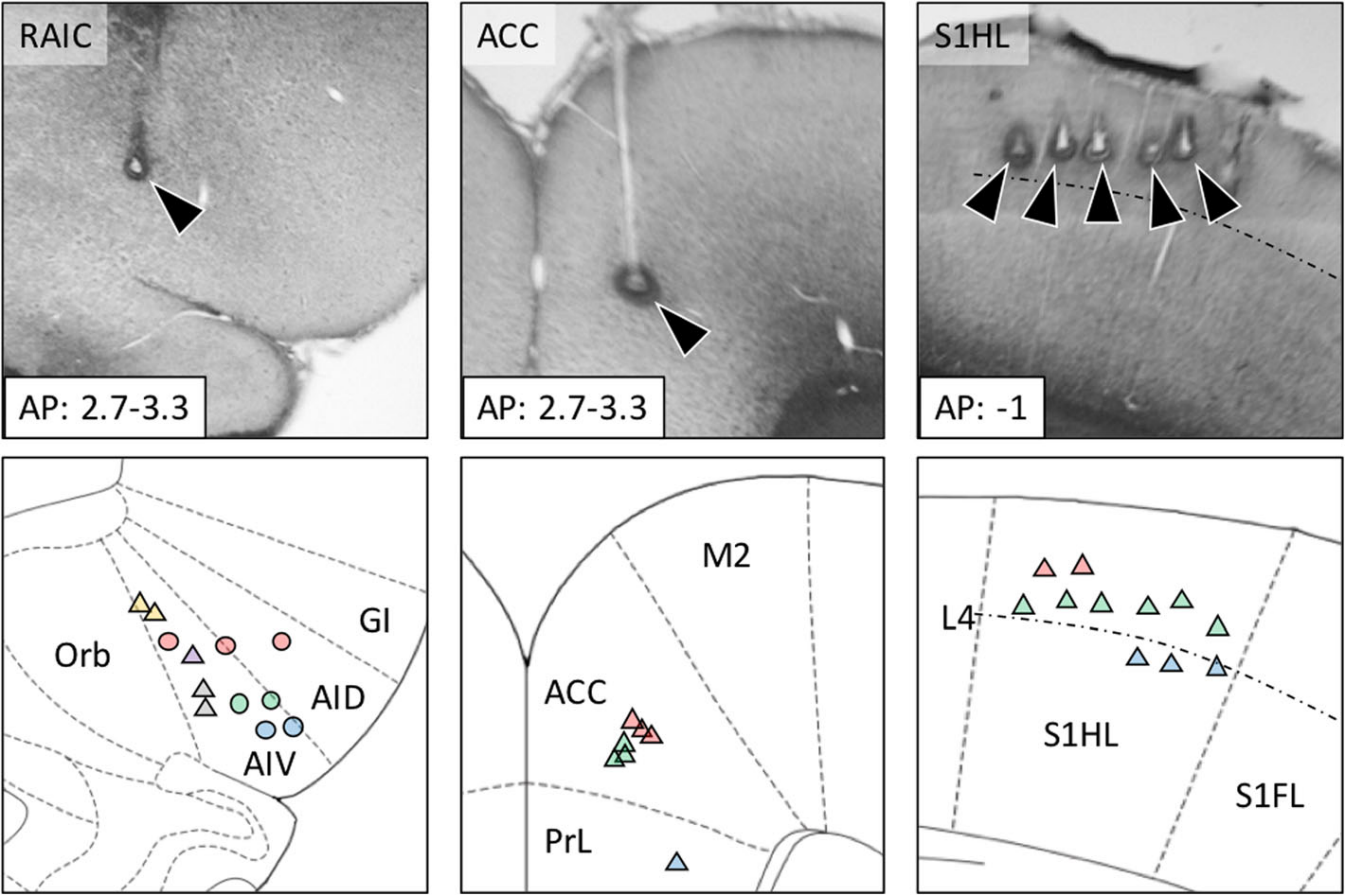
Electrode implantation sites in the RAIC, ACC, and S1. For each brain area, the represented photomicrographs of the histological section are shown in the upper panels. The recording sites were determined by electrolytic lesions at the end of the microwire tracks (arrowheads). The summaries of all recording sites for the unit recordings are shown in the lower panels. The electrode target on the contralateral side is labeled with triangular symbols (▲) and those on the ipsilateral (left) side of the RAIC is labeled with circles (●). Each color represents an electrode site for one individual. Numeral unit for the anterior–posterior length from the bregma (AP) is in millimeters

**Fig. 7:**
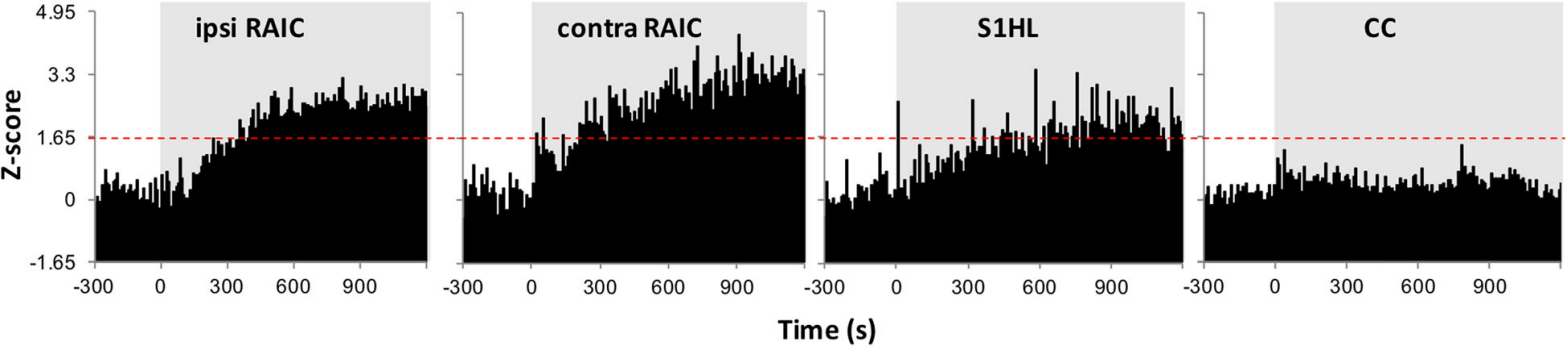
Unit responses during SNI surgery. SNI nerve transection induced a sustained increase in spike activity in the bilateral RAIC and contralateral S1HL are of the injured hind limb, whereas the CC exhibited no significant changes. The spike rates of each unit were converted to z-scores by using the following equation: (FRi – FRb)/SDb, where FRi is the firing rate in the *i*th bin of the recording period (5 s for each bin) and FRb and SDb represent the mean firing rate and standard deviation of firing rates before SNI nerve transection, respectively. The group z-scores were averaged from the mean z-scores of individuals. The spike activities under the resting baseline condition from − 300 to 0 s are shown. SNI nerve transection was performed at time = 0 and followed by 1200 s of post-SNI recording (gray area). The dashed red line shows the threshold of *P* < .05 (total unit number/number of rats in each group: 25/3 in the ipsilateral RAIC, 23/3 in the contralateral RAIC, 22/3 in the S1HL area, and 30/3 in the CC)

Multi-channel, multiple single unit recording experiments were performed in a separate series of experiment outside the MR chamber to independently examine the neuronal responses in selected forebrain regions to nerve transection. These brain regions included the bilateral RAIC, S1HL and ACC. The locations of the recording sites are shown in Fig. 6. The multiunit recording of the responses to nerve transection in the bilateral RAIC and S1HL area confirmed the sustained enhancement of neural activity (more than 20 min after transection) in both brain regions (Fig. 7).

**Fig. 8:**
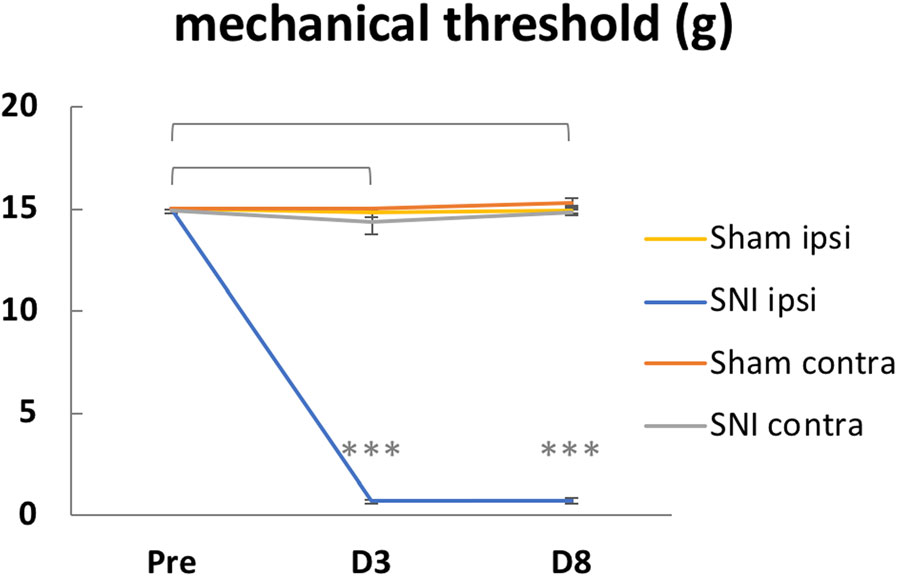
Behavioral test of mechanical allodynia in SNI rats. The 50% withdrawal threshold of the ipsilateral hind paws of SNI rats (*n* = 18) decreased significantly in the first 3 days after SNI surgery and remained low for at least 8 days in total (***: *P < 0.005*). The withdrawal thresholds of the contralateral hind paw in the SNI group and bilateral hind paws in the sham group (*n* = 20) showed no significant changes after surgery. Pre: presurgery

After fMRI, the rats recovered from anesthesia. The 50% withdrawal threshold of the ipsilateral hind paw was tested on day 3 and day 8 after the fMRI experiment. The thresholds were significantly lower on day 3 and day 8 when comparing with pre-experiment baseline (Fig. 8). This indicated our newly designed nerve cutting device did successfully produced SNI rats.

**Fig. 9:**
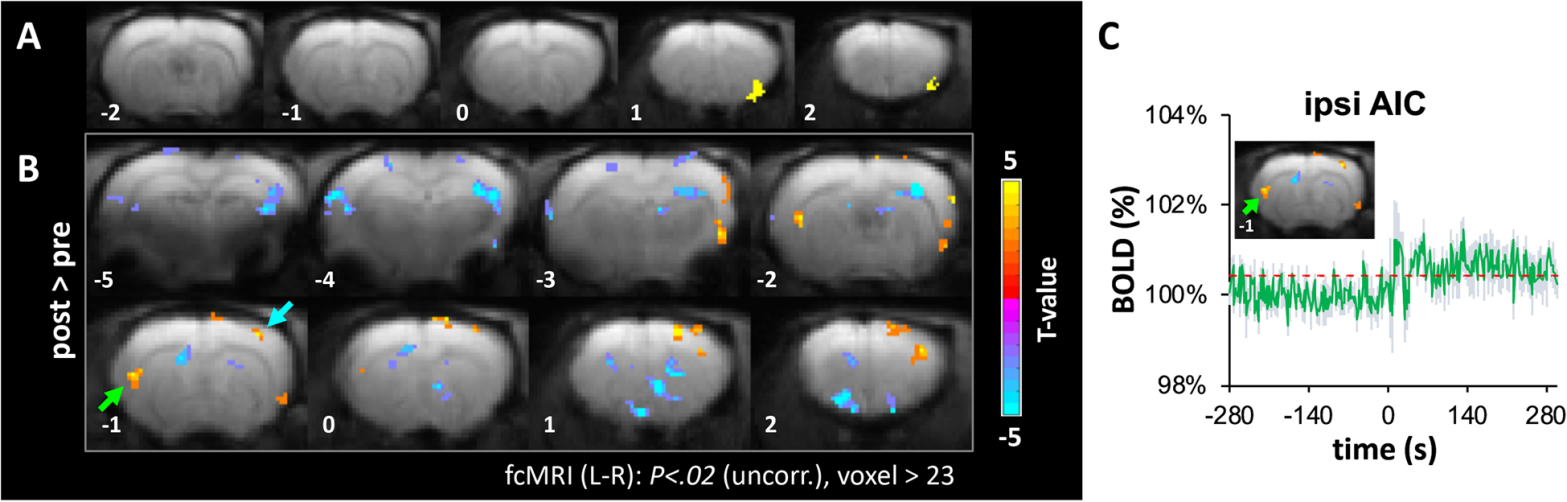
Comparison of the RAIC BOLD-functional network between pre- and post-SNI. In sub-Figs. a and b, coronal slices of the rat brain are shown from the left side the most caudal level to the most rostral level on the right side. In B, statistical t-maps are shown in 8 consecutive coronal slices, 1 mm each, from the most caudal level (5 mm caudal to the bregma, upper-left corner) to the most rostral (2 mm rostral to the bregma, lower-right). (**a**) The yellow area shows the seeding ROI at coronal sections “Introduction” mm and 2 mm rostral to the bregma for generating the RAIC functional network in Fig. 9b, which was defined by the brain activity in Fig. 5a within the RAIC area. (**b**) Comparison map of the RAIC functional network between pre- and post-SNI (warm color: post > pre, cold color: post < pre, significance threshold: *P < .02* with cluster size > 23 voxels). To get the comparison map, EPI images acquired within the first 4 min and the final 4 min of the fMRI experiments (Fig. 1a) were used to generate the RAIC functional network maps of the pre- and post-SNI, respectively. These correlation maps were transformed into z-statistical parametric maps for the pair-t test between the pre- and post-SNI. The BOLD-functional connectivity between the contralateral RAIC (yellow in (A)), contralateral S1 (blue arrow), and ipsilateral AIC (green arrow) were enhanced significantly after SNI relative to the pre-SNI baseline. (**c**) The average BOLD signal time course of the ipsilateral AIC exhibited sustained activity immediately after SNI. The ROI of the ipsilateral AIC was derived from Fig. 9a (green arrows in the insets). The standard errors are shown in light blue. The dashed red line indicates the boundary of |z-score| = 2.33 (*P* < .01)

The significantly enhanced activity immediately after SNI in the RAIC raised the question whether a change in the RAIC functional connectivity network occurred. The EPI images acquired within the first 4 min and the final 4 min of the fMRI experiments were used to generate correlation maps of the pre- and post-SNI groups, respectively. The correlation maps seeded in the contralateral RAIC (the RAIC ROI is shown in Fig. 9a) were transformed into z-statistical parametric maps for comparison between the pre- and post-SNI groups. We found that the functional connections between the contralateral RAIC, contralateral S1, and ipsilateral AIC were enhanced immediately after SNI (Fig. 9b). Upon using the ipsilateral AIC cluster as the ROI to observe the signal time course during the fMRI experiment, we found that the ipsilateral AIC exhibited sustained activation immediately after SNI (Fig. 9c). These data imply that the sustained activities in the ipsilateral AIC observed during the MEMRI experiment may have started at the time point immediately after SNI.

### Subacute changes of functional brain activities and connectivity probed with MEMRI 1 day and 8 day after SNI

In addition to using BOLD-fMRI to study the acute brain responses to nerve transection, we used MEMRI to study the 24-h cumulative brain activity on Day 1 after SNI surgery (Fig. 1b). We found reduced activity in the contralateral S1HL area and enhanced activity in the ipsilateral AIC in the SNI surgery group compared to the naïve and the sham surgery groups (Fig. 4b). To determine whether these brain activity changes would be long-term, we conducted MEMRI to study the brain activity on Day 8 after SNI surgery. We found ipsilateral AIC and RAIC activities in the SNI group were higher than their counter parts of the sham group (Fig. 4c) on the Day 8. The MEMRI results showed that only in ipsilateral AIC and RAIC, there were long-term changes. Activities in the S1HL area and CC were not significantly changed on day 1 and day 8 after SNI surgery (Fig. 4b and c); in contrast with the fMRI results (Fig. 4a) and unit recording results (Fig. 7) immediately after SNI surgery. These data showed that SNI surgery induced sustained activation in the IC, and the enhanced activity lasted for at least 8 days.

**Fig. 10:**
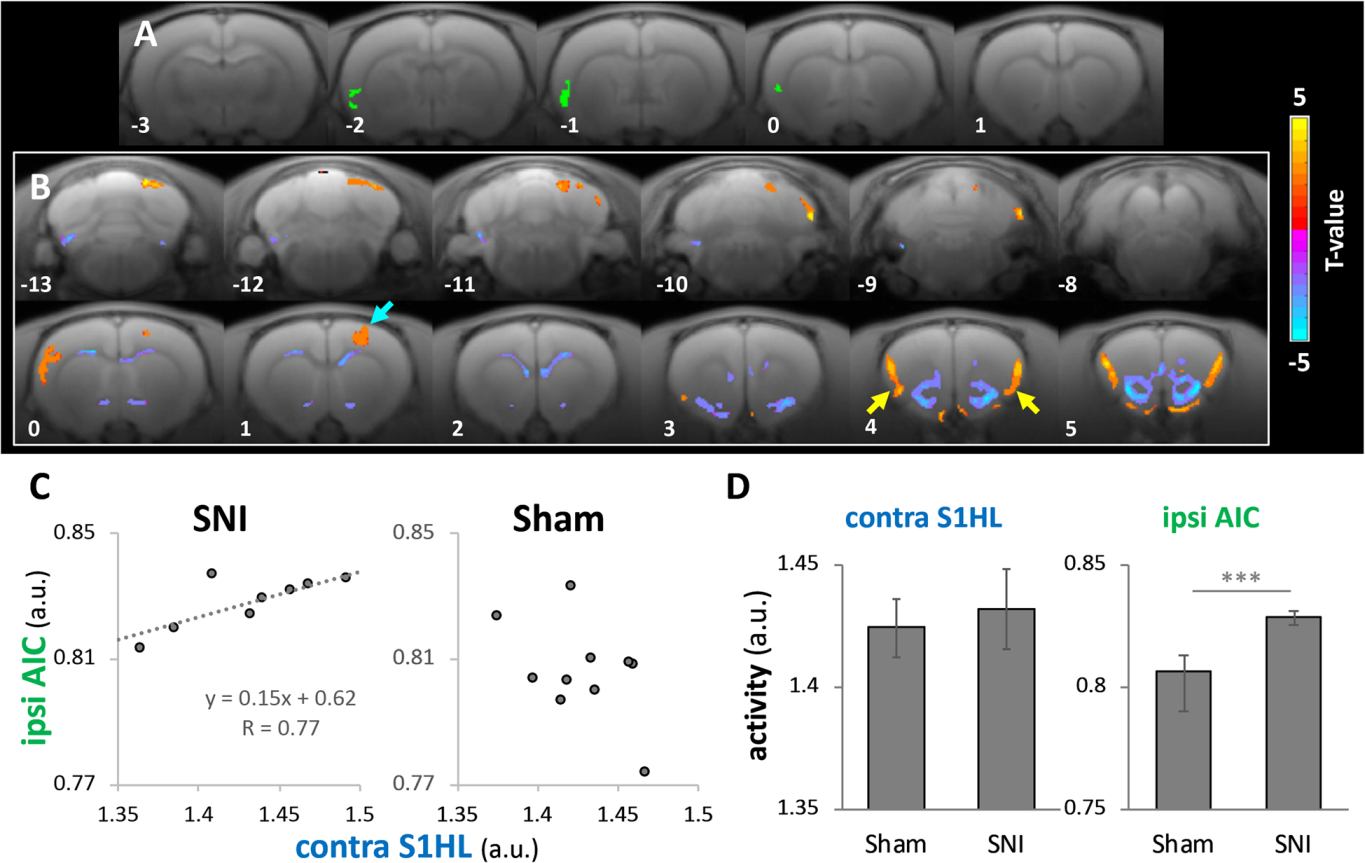
MEMRI-functional connectivity seeded from ipsilateral AIC of neuropathic pain rats on Day 8 after SNI. In sub-Figs. a and b, coronal slices of the rat brain are shown from the left side the most caudal level to the most rostral level on the right side. In B, statistical t-maps are shown in 12 representative coronal slices, from the most caudal level (13 mm caudal to the bregma, upper-left corner) to the most rostral (5 mm rostral to the bregma, lower-right). (**a**) The green area shows the seeding ROI at coronal sections “Methods” mm, 1 mm and 0 mm caudal to the bregma for the connectivity analysis in Fig. 10b, which was defined by the brain activity in the AIC area shown in Fig. 4c. (**b**) MEMRI-functional connectivity map generated through multiple regression analysis with the inter-subject variability of AIC activations as the regressor. The contralateral S1HL area (blue arrows) and bilateral RAIC (yellow arrows) exhibited significant functional connections with the AIC. (**c**) Activity within the significant area of the contralateral S1HL area (blue arrows) was extracted. Activity in the ipsilateral AIC and contralateral S1HL area are plotted together (sham: *n* = 10; SNI: *n* = 8). The SNI group alone exhibited a significant activity correlation between the ipsilateral AIC and contralateral S1 (*P* < .05). (**d**) Quantitative neural activity in the contralateral S1HL area and ipsilateral AIC. Activity in the ipsilateral AIC was significantly higher in the SNI group than in the sham group (*P* < .005), whereas no difference in contralateral S1HL activity was observed between these two groups

The observation of a continuous increase in ipsilateral AIC activity after SNI surgery implies that long-term plasticity changes may be triggered within the ipsilateral AIC-related networks. To determine how the ipsilateral AIC interacted with the other brain areas in the SNI neuropathic pain rats, we used the MEMRI images acquired on Day 8 after surgery to extract the ipsilateral AIC activity of various subjects and used these quantified activities as regressors to search for other brain areas with synchronized activity fluctuations. The ROI of the ipsilateral AIC was defined as the area of significant activation in the statistical map in Fig. 4C (SNI > sham and naïve, Day 8) that overlapped within the anatomical AIC region. The green area in Fig. 10A represents the seeding ROI. We found significant cooperation between the ipsilateral AIC versus bilateral RAIC and S1HL area contralateral to the side with nerve injury in the SNI rats (Fig. 10b and c), consistent with our fMRI data (Fig. 9b). This functional correlation was not observed among the sham rats (Fig. 10c); however, although the ipsilateral AIC activity was significantly higher in the SNI rats than the sham rats (*P = .0038*), the contralateral S1HL activity was similar in both groups (Fig. 10d), indicating that under similar somatosensory input conditions, the ipsilateral AIC exhibited an increase in sensory-related activity only in the SNI rats.

**Fig. 11:**
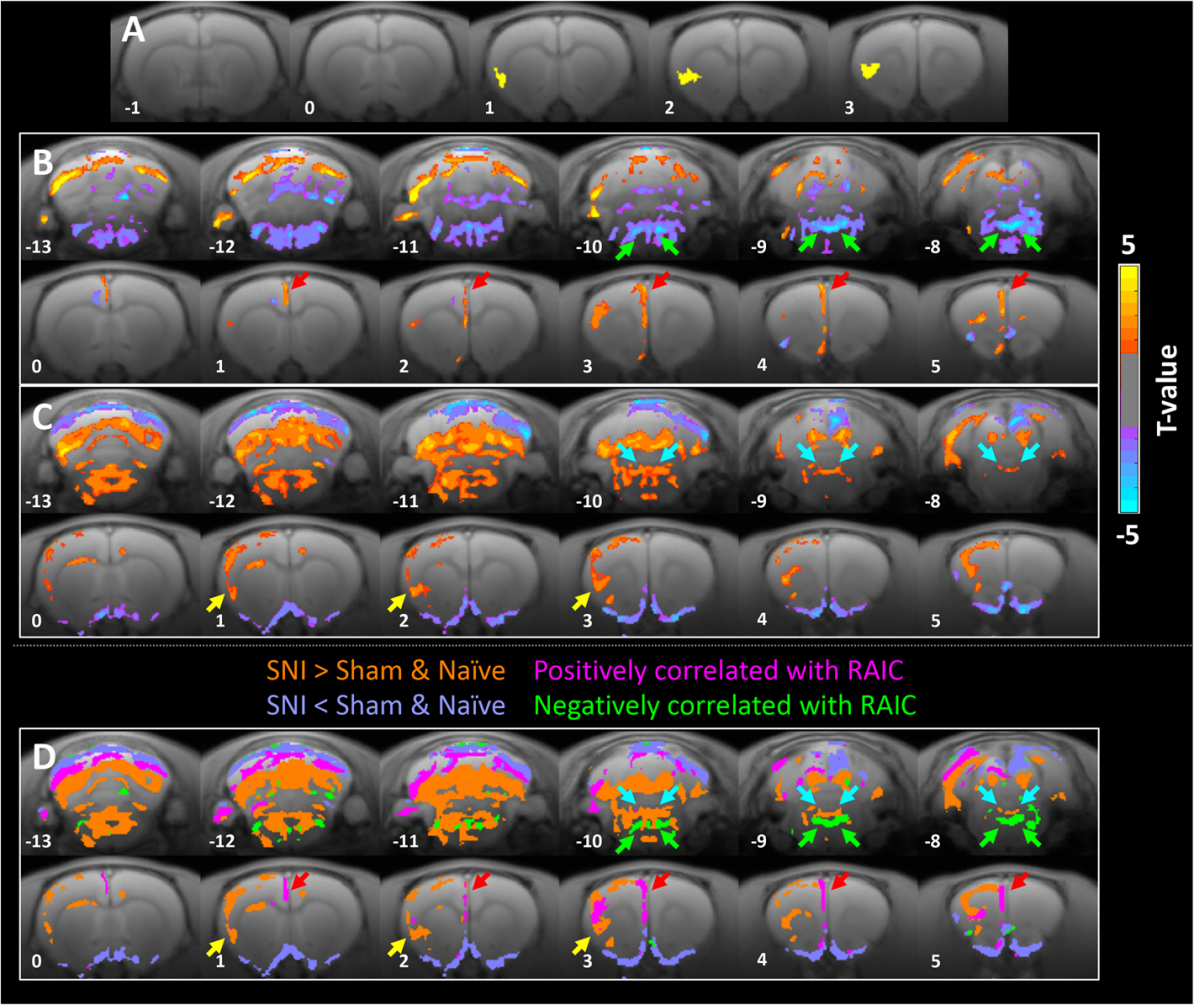
MEMRI-functional connectivity seeded from ipsilateral RAIC of neuropathic pain rats on Day 8 after SNI. In sub-figures, coronal slices of the rat brain are shown from the left side the most caudal level to the most rostral level on the right side. In **b**, **c** and **d**, statistical t-maps are shown in 12 representative coronal slices, from the most caudal level (13 mm caudal to the bregma, upper-left corner) to the most rostral (5 mm rostral to the bregma, lower-right). (**a**) The yellow area shows the seeding ROI at coronal sections “Introduction” mm, 2 mm and 3 mm rostral to the bregma for the connectivity analysis in Fig. 11b, which was defined by the brain activation in the RAIC area shown in Fig. 4c. (**b**) MEMRI-functional connectivity map generated through multiple regression analysis with the intersubject variability of RAIC activations as the regressor. The contralateral ACC (red arrows) and dorsal brainstem (green arrows) areas exhibited significant functional connections with the RAIC (*P* < .05). (**c**) Voxel-wise comparison of brain activity between SNI and sham group rats on Day 8 day after SNI surgery. The SNI group exhibited increased activity in the RAIC (yellow arrows) and dorsal brainstem (blue arrows) areas (*P* < .05). (**d**) Merged image of (**b**) and (**c**). The strong negatively correlated brainstem areas (*P < .003*) derived from (**b**) are denoted in green and mostly located in the LC. The positively correlated brain areas in (**b**) are denoted in magenta and located around the LC without having a colocalized area

We performed a regression analysis using the signal fluctuations extracted from significant clusters in the ipsilateral RAIC area in Fig. 4c (SNI > sham and naïve, D8). The yellow area in Fig. 11a denotes the seeding ROI. We observed a positive correlation between the ipsilateral RAIC and superficial layer of the ACC and prelimbic areas (Fig. 11b). Notably, the ipsilateral RAIC correlated strongly with the bilateral locus coeruleus (LC) (Fig. 11b) and enhanced activity in the peri-coeruleus regions such as the parabrachial nucleus and nucleus raphe magnus (Fig. 11c). To identify the relative location between the negatively correlated LC and activated peri-coeruleus regions, we overlapped Fig. 11b and c (Fig. 11d). The strong negatively correlated areas are denoted in green (*P* < .003) and the positively correlated areas are shown in magenta (Fig. 11d). Because the LC contributes to descending pain modulations, these results imply that the descending pain modulation pathway may be involved in the development of neuropathic pain in the SNI model.

## Discussion

This study combined functional brain imaging and electrophysiological recording methods to map sustained brain activation regions during early SNI neuropathic pain development in rats. We found nerve injury induced immediate and sustained activity increase in the bilateral ICs and contralateral S1. The elevated activity in the ipsilateral IC was long-lasting and could be observed on Days 1 and 8 after SNI. Plasticity changes in functional connectivity among the ipsilateral AIC, contralateral S1HL, and bilateral RAIC were observed during the first few days of nerve injury. In addition, the enhanced functional connectivity between the RAIC and LC that spread to the brainstem implied that the descending pain modulation system may be involved in the development of neuropathic pain.

Many functional brain imaging studies of neuropathic pain have been conducted, most of which involved humans with well-established chronic pain. Such studies have focused on the brain responses to various stimulations [36–40] and various brain network patterns [41–43]. However, how nerve injury causes neuropathic pain chronification remains unresolved. Although central sensitization at the spinal level caused by peripheral nerve injury has been reported as activation-dependent [44–47], how nerve injury initially affects brain activity has not been mapped using functional brain imaging methods. Because nerve transection directly activated the peripheral nerve, our findings of brain activation pattern through SNI are similar to those reported in previous studies that use direct peripheral nerve stimulation on rats [48]. The novelty of the current study is the finding that activity in the bilateral ICs, CC, and S1 increased tonically after nerve transection. Such sustained activity may provide a condition for brain sensitization [11, 12].

Some researchers postulate that chronic pain is the result of nociceptive memory in the brain [2, 49, 50]. The center part of this hypothesis is that, similar to memory consolidation, neuropathic pain chronifies inside the brain through a period of sustained high frequency inputs. We tested this hypothesis using resting fMRI, MEMRI and electrophysiological recording experiments. BOLD-fMRI enables the detection of evoked neural activity on the basis of the neurovascular coupling [51–54], however, it is difficult using BOLD-fMRI to quantify the basal neural activity. Although a resting-state fMRI design can be employed to observe large-scale networks inside the brain [55], determining whether activity in a specific brain area is high or low over long periods is difficult. By contrast, MEMRI enables the mapping of cumulative brain activity in free-moving animals over relatively long periods of 3–24 h [24–26, 33, 56, 57]. Furthermore, a recent study used inter-subject variability of brain activity to analyze functional networks during memory consolidation [26]. In the present study, a similar strategy was employed to investigate neuropathic pain chronification. Rather than using the average signals within the atlas-defined ROIs, we used the average signals within the activation cluster as regressors to assess voxel-wise functional connections. This is because we assumed that only parts of the anatomically defined brain regions exhibited abnormal functional connections; for example, we observed that only part of the contralateral S1HL exhibited plasticity change in functional connectivity with the AIC, possibly because a portion of the right S1HL has been deprived of its input from the tibial and common peroneal nerves.

In many studies, the IC has been consistently activated in various pain paradigms. Some studies have suggested that plasticity in the IC is crucial to the maintenance of neuropathic pain [58–60]. However, the IC is involved in various sensory modalities, as well as motor and emotional functions with complex connections with various brain areas [61]. Craig suggested that the IC is a multimodal homeostatic or interoceptive integration area [62]. Its multimodal input property enables it to serve as a multimodal magnitude estimator or salience detector [61, 63]. Some studies have found that the IC encodes pain intensity [64, 65]. However, parcellation of the IC into a pain magnitude estimator or salience detector remains controversial [66, 67]. The IC is currently considered a multidimensional integration site for pain [68]. Recent studies have shown that people with IC lesions can rate the magnitude of evoked acute pain. Because the S1 has been reported to encode pain intensity [69, 70], it may serve as a redundancy system for magnitude estimation [66]. In the present study, we found the ipsilateral AIC exhibited consistently enhanced activity after SNI surgery; and the functional connection between the AIC with the contralateral S1HLalso enhanced under the neuropathic pain condition. These data thereby imply that a plasticity change of the pain magnitude estimation standard may have occurred in the SNI neuropathic pain rats.

Peripheral nerve injury induces S1 reorganization and causes the input-deprived cortex to be occupied by neighborhood expansion [71]. Our data showed that activity within the S1HL decreased on Day 1 after SNI surgery but recovered to a similar level as that of the sham group by Day 8. This recovery may be the result of cortical reorganization and the expanded representation of the neighboring nerves. The association between S1 reorganization and neuropathic pain development is not yet understood. Furthermore, we found the S1HL area began to cooperate with the AIC under the neuropathic pain condition; however, the overall S1HL activity did not differ between the SNI and sham groups. In our previous study, we observed no variations in S1HL responses to von Frey hair stimulation between SNI and naïve rats [34]. Whether and how the S1 is involved in allodynia is the subject of debate. Some studies on people with neuropathic pain have reported increase activity in the S1 with allodynic stimulations [40, 72, 73], whereas others with similar experimental designs have not observed any variations in S1 response [37, 74]. Nevertheless, our data shows the enhanced functional connection between the reorganized S1HL area and AIC in SNI neuropathic pain rats, thereby shedding new light on the association between the S1 and neuropathic pain development.

In this study, the RAIC exhibited sustained activity in response to SNI and strongly enhanced activity on Day 8 after SNI. A previous anatomical study revealed that the RAIC received considerable input from the centrolateral thalamus (CL) and mediodorsal thalamus (MD) [75], which showed strong activity in response to SNI in the fMRI result. The CL and MD are major sites of termination for the spinothalamic tract [76–79] and are implicated in the sensorimotor integration of nociceptive processing [80]. Our data showed that after SNI, RAIC activity was strongly and negatively correlated with the LC and several brainstem areas, whereas the peri-coeruleus region exhibited enhanced neural activity. According to a previous anatomical study, the RAIC projects to the peri-coeruleus region, rostroventral medulla, lateral hypothalamus, parabrachial area, dorsal raphe, and periaqueductal gray [75], all of which have been directly linked to brainstem-mediated descending modulation in spinal nociceptive neurons [81–87]. However, a previous study demonstrated that the activation of RAIC projection neurons can cause hyperalgesia in a top-down manner [88]. Two groups of RAIC projection neurons respectively act upon two independent subcortical nuclei to modulate the nociceptive threshold. The first group projects to the gamma-aminobutyric acid (GABA) interneurons in the peri-coerulear zone, and these GABAergic interneurons inhibit the LC neurons and affect the noradrenergic bulbospinal projections. The second group projects to the amygdala and causes hyperalgesia when activated [88]. Therefore, our data may imply a plasticity change in the pain threshold through a top-down modulation from the RAIC after SNI.

The limitation of the present study is the relatively small number of rats used in the BOLD fMRI and its supplementary electrophysiological studies. The classical spared nerve injury model transected the 2 larger branch of the sciatic nerve and leaves only the minor sural nerve intact. This is a major trauma to the rat. SNI is a very robust neuropathic pain model with minimal variations. This has been described by Decosterd & Woolf in the abstract of their original paper: “*The spared nerve injury model results in early (<24 h), prolonged (>6 months), robust (all animals are responders) behavioral modifications.*” [30]. The robustness of the SNI model has been repeated in our lab in several previously published papers [34, 89] and can be clearly observed in the Fig. 8 of the present study. Nevertheless, the small number of animal used prevents a meaningful analysis of possible difference between male and female rats.

In summary, this study constitutes the first experimental observation of the forebrain regions with tonically enhanced activity induced by peripheral nerve injury and the first study to correlate this tonic brain activity with long-term brain plasticity. The SNI-induced plasticity changes in the ipsilateral AIC, bilateral RAICs, and S1 may contributed to the neuropathic pain development through changes in the sensorimotor integration of nociceptive information. Moreover, the negative functional correlation between the RAIC and LC suggested a change in the descending pain modulation system under the condition of neuropathic pain development.

## Abbreviations

ACC: anterior cingulate cortex
AIC: anterior insular cortex
BOLD-fMRI: blood-oxygenation-level-dependent functional magnetic resonance imaging
CC: cingulate cortex
CL: centrolateral thalamus
CPu: caudate putamen
EPI: echo planar imaging
FWHM: full width at half maximum
GABA: gamma-aminobutyric acid
IC: insular cortex
MD: mediodorsal thalamus
MDL: mediodorsal thalamic nucleus, lateral part
MDM: mediodorsal thalamic nucleus, medial part
MEMRI: manganese-enhanced magnetic resonance imaging
RAIC: rostral anterior insular cortex
ROIs: regions of interest
S1: primary somatosensory cortex
S1HL: S1 hind limb
SNI: spared nerve injury
VP: ventral posterolateral thalamic nucleus
